# Enhancing therapeutic outcomes in degenerative lumbar disc disease: a retrospective study on combined autologous PRP injection and celecoxib therapy

**DOI:** 10.3389/fmed.2026.1886181

**Published:** 2026-09-22

**Authors:** Kunpeng Ruan, Hushan Jiang, Tao Meng, Shousong Huo, Guojun Li

**Affiliations:** Department of Orthopedics, Huaihe Hospital, Henan University, Kaifeng, Henan, China

**Keywords:** autologous PRP injection, celecoxib, degenerative lumbar disc disease, functional impairment, inflammatory response, pain relief

## Abstract

**Objective:**

To investigate the therapeutic value of autologous platelet-rich plasma (PRP) injection in patients with degenerative lumbar disc disease (DLDD), and to evaluate its clinical efficacy in alleviating pain, improving lumbar function, modulating inflammatory responses, and enhancing quality of life.

**Methods:**

This retrospective study included 112 DLDD patients treated at our hospital from April 2023 to February 2025. According to the treatment received, patients were divided into two groups. The control group (*n* = 56) received oral celecoxib, while the observation group (*n* = 56) received autologous PRP injection in addition to oral celecoxib. The two groups were compared regarding treatment efficacy, pain response [Visual Analog Scale (VAS)], lumbar function [Japanese Orthopedic Association score (JOA)], disability in daily activities [Oswestry Disability Index (ODI)], inflammatory markers [Toll-like receptor 4 (TLR4), tumor necrosis factor-α (TNF-α), high-sensitivity C-reactive protein (hs-CRP), stromal cell-derived factor-1 (SDF-1)], and incidence of adverse reactions.

**Results:**

The observation group demonstrated significantly superior overall efficacy compared with the control group (total effective rate: 94.96% vs. 82.14%, *P* < 0.05). There were significant differences between groups, over time, and in group-time interaction for VAS, JOA, and ODI scores (*P* < 0.05). At 4 weeks after treatment, TLR4, TNF-α, hs-CRP, and SDF-1 levels in both groups decreased compared with baseline, with greater reductions in the observation group (*P* < 0.05). The incidence of adverse reactions was similar between groups (8.93% vs. 14.29%, *P* > 0.05).

**Conclusion:**

Combining autologous PRP injection with oral celecoxib provides short-term pain relief, functional improvement, and reduction of inflammatory responses in DLDD patients without significantly increasing adverse reactions. However, longer follow-up is required to determine the durability of these effects and the long-term clinical value of PRP-based treatment.

## Introduction

Degenerative lumbar disc disease (DLDD) is one of the most common spinal degenerative disorders causing chronic low back pain and restricted lumbar mobility. Its pathophysiology primarily involves loss of nucleus pulposus hydration, annulus fibrosus degeneration, increased release of inflammatory mediators, and sensitization of nerve endings, leading to reduced lumbar stability and persistent pain (1, 2). With population aging, accelerated lifestyles, and increased prevalence of sedentary or physically demanding occupations, the incidence of DLDD has risen annually, severely impacting patients' quality of life and work capacity, and representing a common challenge in orthopedic clinical management (3). Current clinical strategies for DLDD mainly include non-steroidal anti-inflammatory drugs (NSAIDs), physical rehabilitation, and lumbar traction, which primarily relieve pain but cannot fundamentally reverse disc degeneration or repair local tissue, and symptoms may recur after cessation of therapy (4, 5). Therefore, introducing novel biological therapies as adjuncts to conservative treatment to enhance pain relief, improve functional recovery, and modulate local inflammatory responses has become a focus in orthopedic and rehabilitation research.

Autologous platelet-rich plasma (PRP) is an autologous blood-derived preparation with high concentrations of platelets and multiple growth factors, possessing biological functions such as promoting tissue regeneration, inhibiting inflammation, and repairing soft tissue injuries (6). Currently, autologous PRP injection has been widely applied in burn recovery, knee osteoarthritis, and sports-related soft tissue injuries (7–9). However, clinical evidence for DLDD patients remains limited, with a lack of systematic studies integrating pain scores, functional assessments, and inflammatory biomarkers. Therefore, this study applied autologous PRP injection in combination with conventional oral NSAID therapy (celecoxib) in DLDD patients, assessing pain intensity, lumbar function, disability, and serum inflammatory markers to evaluate clinical efficacy and safety, providing a reference for minimally invasive biological treatment of DLDD.

## Materials and methods

### Study subjects and design

This study was designed as a single-center retrospective controlled study, including 112 patients diagnosed with DLDD who visited the Pain or Spine Surgery departments of our hospital from April 2023 to February 2025. All cases met the diagnostic criteria for degenerative lumbar disc disease (10), based on clinical manifestations (chronic low back pain, aggravated by standing or activity) and imaging findings (MRI showing disc degeneration, reduced signal intensity, T2-weighted protrusion, or annulus fibrosus fissures). This retrospective study involving human participants was approved by the Ethics Committee of Huaihe Hospital, Henan University (Ethics No.: TTZJB2411LC). All enrolled patients provided informed consent for the use of their clinical data for research purposes. Patient privacy and confidentiality were strictly protected, and the study was conducted in accordance with the ethical principles of the Declaration of Helsinki.

### Inclusion and exclusion criteria

Inclusion criteria: (1) Age 30–70 years, any gender; (2) single-level degeneration confirmed by MRI or CT; (3) VAS score ≥4 and disease duration ≥3 months; (4) poor response to initial conservative treatment, voluntarily accepting PRP injection or medication therapy; (5) complete clinical and follow-up data available. Exclusion criteria: (1) Disc herniation with severe nerve root compression requiring surgery; (2) spinal canal stenosis, spondylolisthesis, spinal infection, tumor, or active tuberculosis; (3) autoimmune diseases such as gout or rheumatoid arthritis; (4) hematological disorders, coagulation abnormalities, or severe liver/kidney dysfunction; (5) pregnancy, lactation, or recent use of other biological therapies; (6) allergy to celecoxib or blood products; (7) incomplete follow-up data or withdrawal from the study.

### Grouping and treatment protocols

According to treatment type, the 112 patients were divided into two groups: Control group (*n* = 56): Oral celecoxib capsules (Pfizer, China, Approval No. J20030099), 200 mg twice daily for 4 weeks, avoiding other NSAIDs or corticosteroids. Observation group (*n* = 56): Received the same oral celecoxib regimen as the control group, combined with autologous PRP injection. PRP preparation and injection procedure: For patients in the observation group, 40 mL of peripheral venous blood was collected in the fasting state in the morning using sterile anticoagulant tubes. PRP was prepared under aseptic conditions by trained personnel using the same centrifugation protocol throughout the study period. A two-step centrifugation method was applied: the first spin was performed at 1,500 rpm for 10 min to separate red blood cells, followed by a second spin at 3,500 rpm for 15 min to obtain the platelet-rich fraction. Approximately 5 mL of PRP was obtained from each preparation, of which 3 mL was used for injection. Before injection, the PRP was visually inspected to exclude samples with obvious hemolysis, clot formation, or contamination, and the preparation was used immediately after centrifugation. To reduce procedural variability, all PRP procedures were performed using the same equipment, centrifugation parameters, injection volume, and aseptic operating standards. Patients were placed in the prone position, and the affected lumbar segment was localized under C-arm fluoroscopy or ultrasound guidance. A 3 mL PRP injection was administered into the paraspinal interspace under aseptic conditions. Injections were repeated every 2 weeks for two sessions. After injection, patients rested in a supine position for 30 min while local reactions were observed. All patients were advised to avoid strenuous bending or lifting and were guided on lumbar and back muscle exercises and rehabilitation.

### Observation indicators

Given the retrospective nature of the study, the follow-up schedule was based on routinely documented clinical visits. The 4-week post-treatment follow-up was selected because it represented the most consistently available follow-up interval after completion of the initial treatment cycle. Therefore, the present study mainly evaluated short-term clinical efficacy, inflammatory response, and early safety rather than long-term durability.

1. Treatment efficacy: Classified as remarkable, effective, or ineffective. Remarkable: pain nearly disappeared, function normal; effective: pain significantly reduced, function improved; ineffective: symptoms not significantly improved. Total effective rate = (remarkable + effective)/total × 100%.
2. Pain assessment: Visual Analog Scale (VAS) (11), 0–10 points, higher scores indicate greater pain. Evaluated at before treatment, 4 weeks after treatment, and 4 weeks follow-up.
3. Lumbar function assessment: Japanese Orthopedic Association (JOA) score (12), including subjective symptoms (9 points), clinical signs (6 points), restriction of daily activities (14 points), and bladder function (−6–0 points); total score 0–29, higher scores indicate better lumbar function. Assessed at before treatment, 4 weeks after treatment, and 4 weeks follow-up.
4. Disability assessment: Oswestry Disability Index (ODI) (13), 10 items (pain, self-care, lifting, walking, sitting, standing, sleeping, sexual activity, social life, travel), each scored 0–5; total score 0–50, higher scores indicate greater disability. Assessed at before treatment, 4 weeks after treatment, and 4 weeks follow-up.
5. Inflammatory markers: 5 mL fasting peripheral venous blood was collected, centrifuged at 3,000 rpm for 10 min to separate serum, and ELISA was used to measure TLR4, TNF-α, hs-CRP, and SDF-1 levels. Kits were purchased from Tianjin Asier Biotechnology Co., Ltd. Assessed at before treatment and 4 weeks after treatment.
6. Adverse reactions: Recorded events including local redness, tenderness, fever, dizziness, gastrointestinal discomfort, etc., and calculated the incidence.

### Statistical analysis

GraphPad Prism 8 was used for graphing; SPSS 22.0 for data analysis. Continuous data were assessed for normality using the Shapiro-Wilk test, expressed as ($\overset{-}{x}\pm s$). Paired *t*-tests were used for within-group comparisons, independent *t*-tests for between-group comparisons, and repeated measures ANOVA (RM-ANOVA) for different time points within groups; simple effect analysis was performed if interaction existed. Categorical data were expressed as n (%) and analyzed using χ^2^ or Fisher's exact test. Two-sided tests were used, with α = 0.05; *P* < 0.05 was considered statistically significant.

## Results

### Comparison of baseline characteristics between the two groups

As shown in Table 1, there were no significant differences between the two groups in terms of sex, age, body mass index (BMI), disease duration, affected segment, or comorbidities (*P* > 0.05), indicating comparability.

**Table 1:** Comparison of baseline characteristics between the two groups [$\overset{-}{x}\pm s$, *n* (%)].

Characteristic	Control (*n* = 56)	Observation (*n* = 56)	*t/x^2^*	*P*
Sex	–	–	0.145	0.702
Male	31 (55.36)	33 (58.93)	–	–
Female	25 (44.64)	23 (41.07)	–	–
Age (years)	53.47 ± 8.26	54.09 ± 8.14	0.400	0.689
BMI (kg/m^2^)	24.61 ± 2.77	24.73 ± 2.85	0.225	0.821
Disease duration (years)	2.79 ± 1.08	2.94 ± 1.12	0.721	0.472
Affected segment	-	-	0.149	0.698
L3–L4	7 (12.50)	6 (10.71)	–	–
L4–L5	33 (58.93)	35 (62.50)	–	–
L5–S1	16 (28.57)	15 (26.79)	–	–
Comorbidities	–	–	–	–
Hypertension	14 (25.00)	17 (30.36)	0.401	0.526
Diabetes	10 (17.86)	12 (21.43)	0.226	0.634

### Comparison of treatment efficacy between the two groups

As shown in Table 2, the observation group demonstrated significantly better overall efficacy than the control group (total effective rate: 94.96% vs. 82.14%, *P* < 0.05).

**Table 2:** Comparison of treatment efficacy between the two groups [*n* (%)].

Treatment effect	Control (*n* = 56)	Observation (*n* = 56)	*x^2^*	*P*
Remarkable	24 (42.86)	32 (57.14)	-	-
Effective	22 (39.29)	21 (37.50)	-	-
Ineffective	10 (17.86)	3 (5.36)	-	-
Total effective rate	46 (82.14)	53 (94.64)	4.264	0.038

### Comparison of pain response between the two groups

As shown in Figure 1, VAS scores demonstrated significant effects of group (*F* = 15.863), time (*F* = 97.329), and interaction (*F* = 51.762) (*P* < 0.05). Within groups, VAS scores at 4 weeks after treatment and at 4-week follow-up were lower than before treatment, and follow-up scores were lower than 4-week after treatment scores (*P* < 0.05). Between groups, the observation group had lower VAS scores than the control group at both 4 weeks and follow-up (*P* < 0.05).

**Figure 1:**
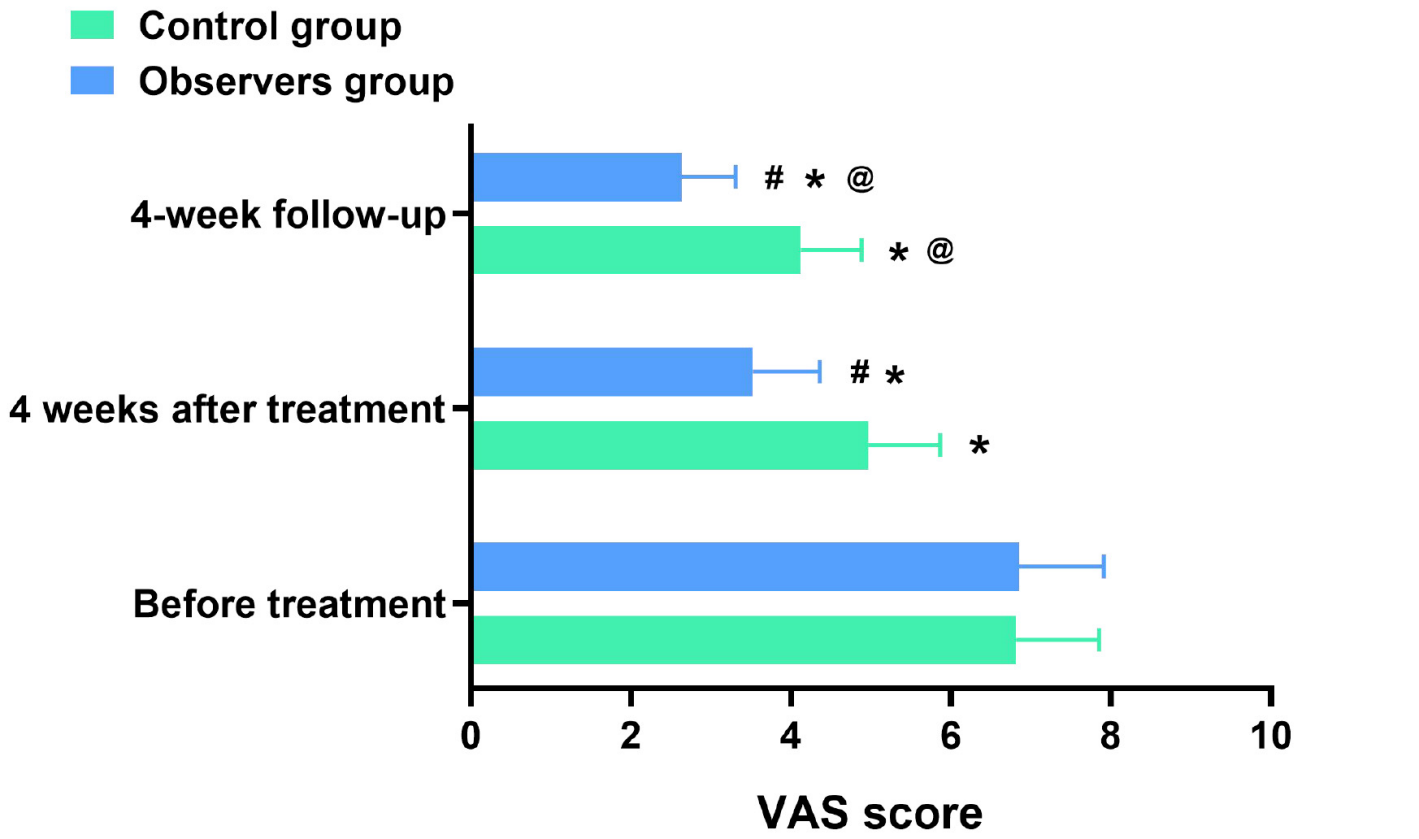
Comparison of pain response between the two groups ($\overset{-}{x}\pm s$, points). Between-group comparison at the same time point, ^#^*P* < 0.05; compared with before treatment within the same group, *P < 0.05; compared with 4 weeks within the same group, ^@^*P* < 0.05.

### Comparison of lumbar function between the two groups

As shown in Figure 2, JOA scores demonstrated significant effects of group (*F* = 17.684), time (*F* = 105.431), and interaction (*F* = 57.813) (*P* < 0.05). Within groups, JOA scores at 4 weeks after treatment and follow-up were higher than before treatment, and follow-up scores were higher than 4-week scores (*P* < 0.05). Between groups, the observation group had higher JOA scores than the control group at both 4 weeks and follow-up (*P* < 0.05).

**Figure 2:**
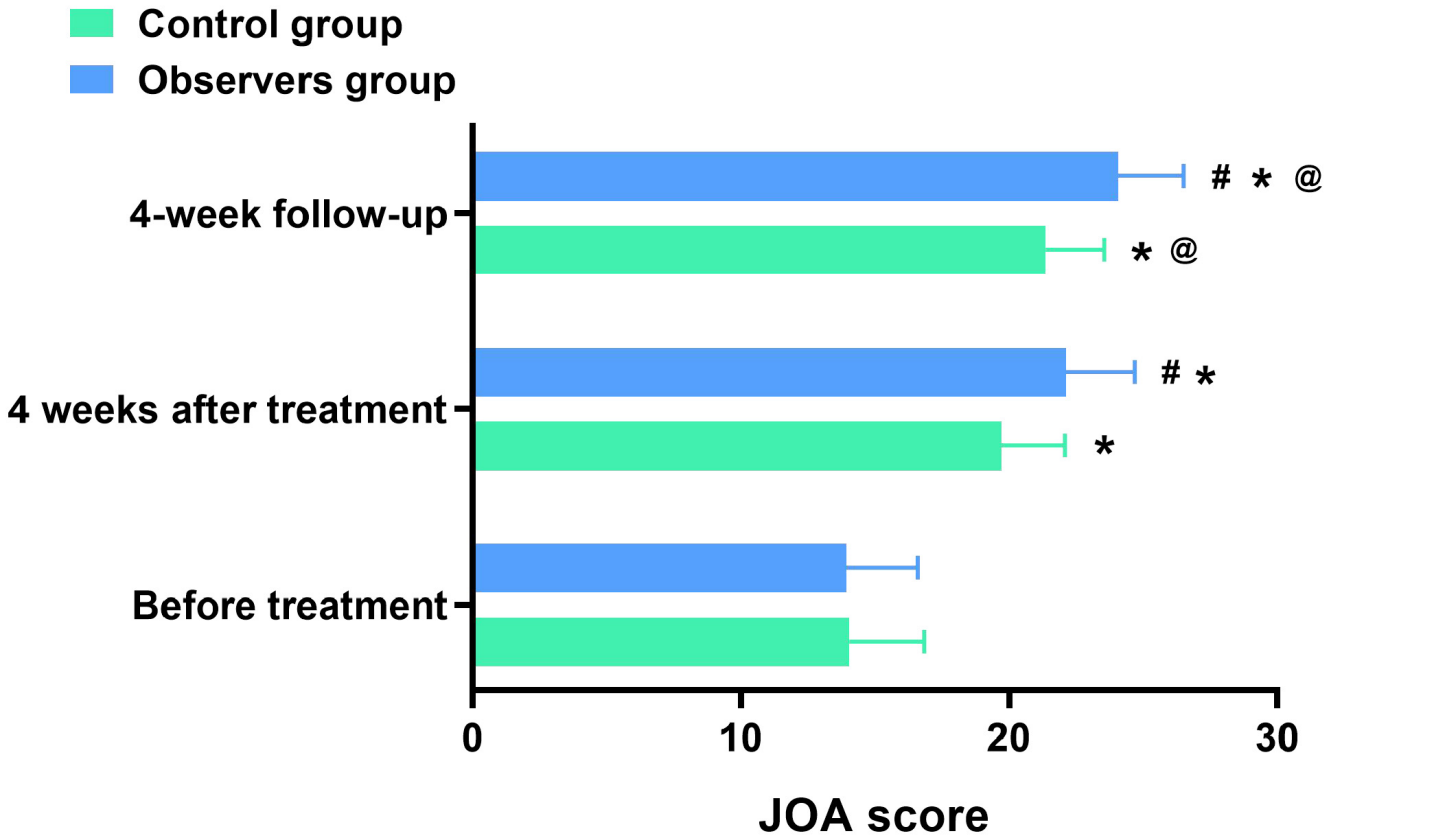
Comparison of lumbar function between the two groups ($\overset{-}{x}\pm s$, points). Between-group comparison at the same time point, ^#^*P* < 0.05; compared with before treatment within the same group, *P < 0.05; compared with 4 weeks within the same group, ^@^*P* < 0.05.

### Comparison of disability between the two groups

As shown in Figure 3, ODI scores demonstrated significant effects of group (*F* = 13.918), time (*F* = 89.743), and interaction (*F* = 47.846) (*P* < 0.05). Within groups, ODI scores at 4 weeks after treatment and follow-up were lower than before treatment, and follow-up scores were lower than 4-week scores (*P* < 0.05). Between groups, the observation group had lower ODI scores than the control group at both 4 weeks and follow-up (*P* < 0.05).

**Figure 3:**
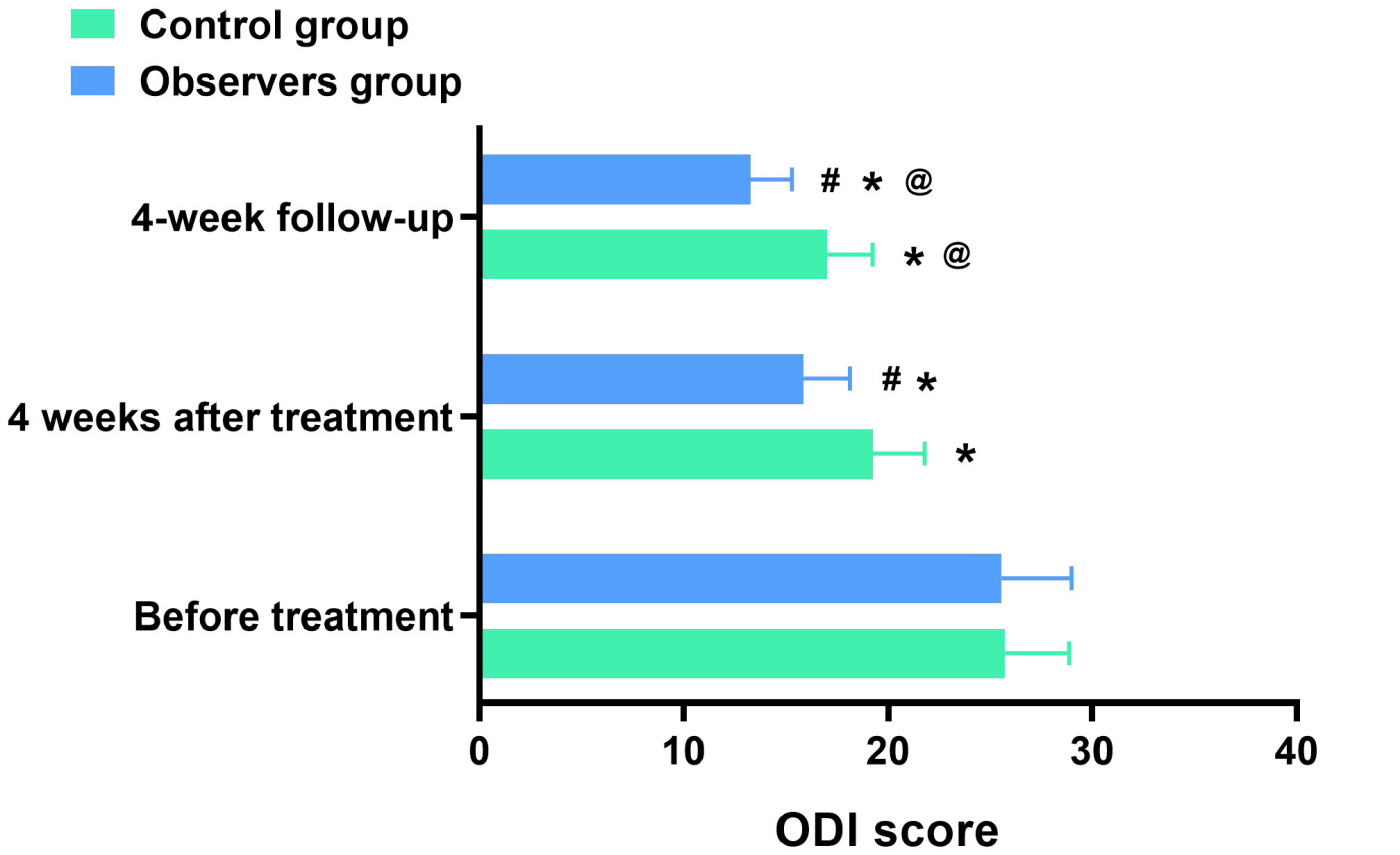
Comparison of disability between the two groups ($\overset{-}{x}\pm s$, points). Between-group comparison at the same time point, ^#^*P* < 0.05; compared with before treatment within the same group, *P < 0.05; compared with 4 weeks within the same group, ^@^*P* < 0.05.

### Comparison of inflammatory markers between the two groups

As shown in Figure 4, at 4 weeks after treatment, TLR4, TNF-α, hs-CRP, and SDF-1 levels decreased in both groups compared with before treatment, with the observation group showing greater reductions than the control group (*P* < 0.05).

**Figure 4:**
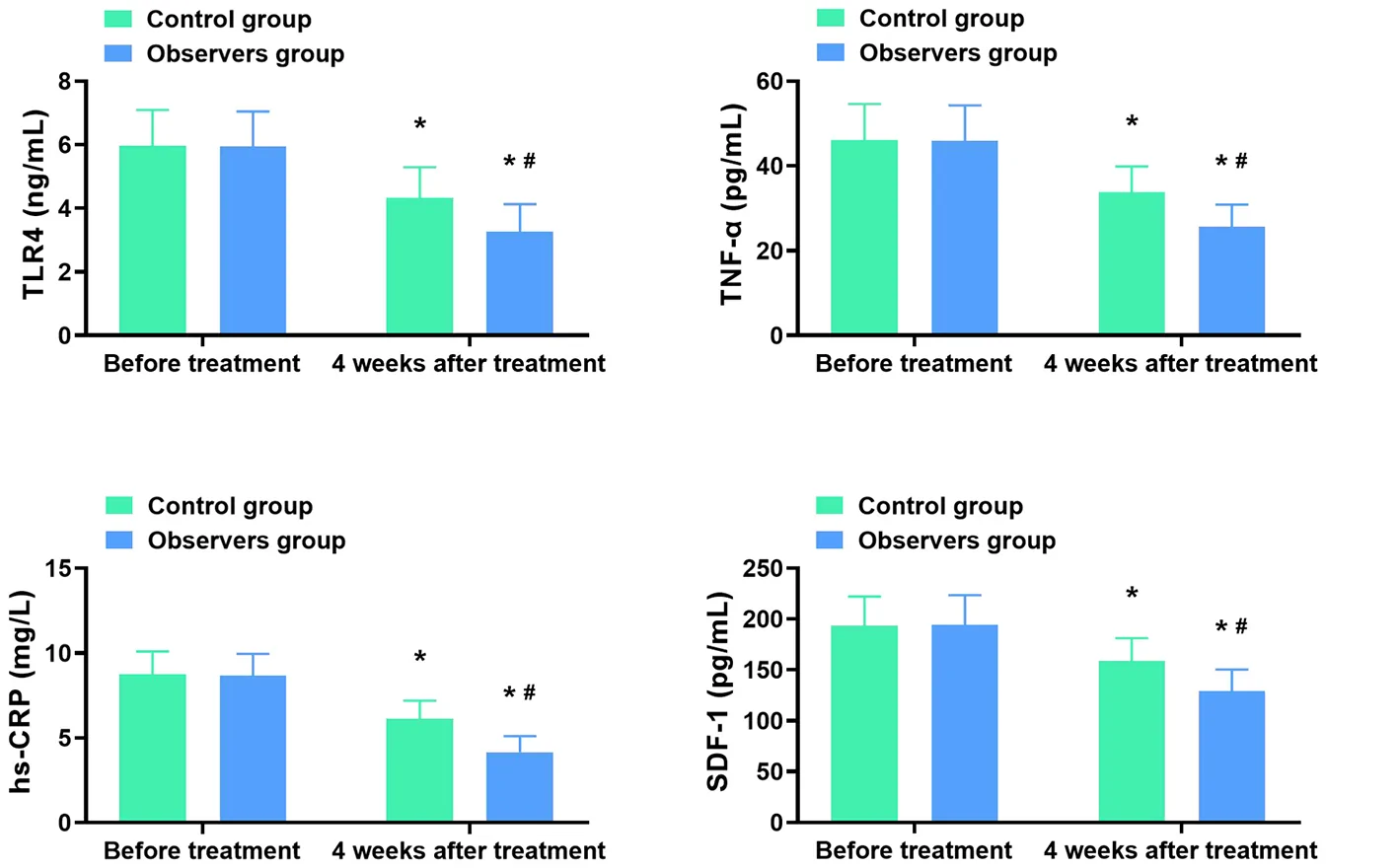
Comparison of inflammatory markers between the two groups ($\overset{-}{x}\pm s$). Between-group comparison at the same time point, ^#^*P* < 0.05; compared with before treatment within the same group, *P < 0.05.

### Comparison of adverse reactions between the two groups

As shown in Table 3, the incidence of adverse reactions was similar between the control and observation groups (8.93% vs. 14.29%, *P* > 0.05).

**Table 3:** Comparison of adverse reactions between the two groups [*n* (%)].

Adverse reaction	Control (*n* = 56)	Observation (*n* = 56)	*x^2^*	*P*
Injection site redness	0 (0.00)	2 (3.57)	-	-
Tenderness	1 (1.79)	1 (1.79)	-	-
Fever	1 (1.79)	2 (3.57)	-	-
Dizziness	1 (1.79)	1 (1.79)	-	-
Gastrointestinal discomfort	2 (3.57)	2 (3.57)	-	-
Total incidence	5 (8.93)	8 (14.29)	0.783	0.376

## Discussion

### Overall efficacy advantage of autologous PRP combined with celecoxib in treating DLDD

The results of this study demonstrated that the overall clinical efficacy of combined PRP and celecoxib treatment was significantly superior to that of oral medication alone, indicating that autologous PRP can substantially enhance therapeutic effects on top of conventional analgesia. Celecoxib exerts short-term analgesic effects by selectively inhibiting the COX-2 pathway and reducing the release of inflammatory mediators such as PGE2 (14), but it does not prevent further intervertebral disc degeneration. PRP is a biologic preparation rich in platelets and multiple bioactive molecules, including platelet-derived growth factor (PDGF), fibroblast growth factor (FGF), transforming growth factor-β (TGF-β), and vascular endothelial growth factor (VEGF), which may participate in inflammatory regulation and extracellular matrix metabolism-related pathways (15). In the present study, the addition of PRP to celecoxib was associated with greater short-term pain relief, functional improvement, and reduction of inflammatory markers. However, because post-treatment MRI-based disc degeneration grading was not evaluated, these findings should be interpreted as clinical and biochemical improvements rather than direct evidence of intervertebral disc regeneration or structural repair. Similar to our findings, Le et al. (16) reported that PRP injection alone improved clinical efficacy and lumbar function in patients with lumbar disc disease to a certain extent. The greater improvement observed in the present study may be related to the local anti-inflammatory and neuromodulatory effects of PRP combined with systemic anti-inflammatory support from celecoxib.

### Mechanistic insights into pain relief

Chronic low back pain is the most prominent clinical manifestation of DLDD. This study showed that VAS scores at 4 weeks after treatment and at 4-week follow-up decreased more significantly in the observation group than in the control group, suggesting a more pronounced overall analgesic effect of PRP. Studies (17) indicate that DLDD-related pain is not only associated with disc structural degeneration and mechanical stress but also closely linked to local inflammation-induced sensitization of nerve endings. PRP contains multiple anti-inflammatory and repair factors that can suppress the expression of inflammatory cytokines such as IL-1β and TNF-α, thereby blocking sensitization processes caused by the continuous release of pain mediators (18). Moreover, PRP can regulate the expression of neuropeptides such as Substance P and CGRP, reducing stimulation of nociceptive fibers and increasing pain thresholds (19). Clinical studies by Zitsch et al. (20) demonstrated that PRP treatment significantly lowered serum PGE2 levels and inhibited inflammatory signaling pathway activation, resulting in a notable VAS reduction, although the magnitude of improvement was still limited. In this study, the combination of PRP injection and systemic celecoxib treatment allowed multi-level inhibition of the COX-2 pathway (21), potentially further enhancing pain control, leading to more pronounced VAS reduction. Therefore, PRP offers unique advantages in local anti-inflammation and neural modulation, and its combination with anti-inflammatory drugs can achieve additive analgesic effects.

### Association between lumbar function improvement and inflammatory factor regulation

JOA scores increased significantly over time, with the observation group consistently outperforming the control group, indicating that PRP treatment substantially benefits lumbar function recovery. Mechanistically, functional impairment arises not only from structural degeneration but also from persistent local inflammation. This study showed that TLR4, TNF-α, hs-CRP, and SDF-1 levels decreased more significantly in the observation group, suggesting that PRP can improve the local microenvironment through multi-target modulation of inflammatory pathways. TLR4, as an innate immune recognition receptor, is persistently activated in DLDD, triggering NF-κB cascades and promoting IL-1, TNF-α, and MMP-3 release, accelerating matrix degradation (22). PRP, rich in bioactive factors such as TGF-β and IGF-1, may participate in the regulation of TLR4/NF-κB-related inflammatory pathways and extracellular matrix metabolism (23). However, because post-treatment structural imaging outcomes were not assessed, this mechanism remains inferential and should be considered a possible explanation for the observed inflammatory marker changes and functional improvement rather than direct evidence of disc repair. SDF-1, as a chemokine, promotes inflammatory cell recruitment and amplifies aseptic inflammation (24); its significant reduction in the observation group suggests that anti-inflammatory cytokines in PRP may inhibit macrophage infiltration, stabilizing the local microenvironment. Yan et al. (25) also demonstrated that PRP suppresses TGF-β1 and MMP-3 expression while enhancing disc cell metabolic activity, consistent with the reductions in inflammatory markers observed in this study, supporting the possibility that PRP may be associated with modulation of microinflammation in DLDD. Nevertheless, the present study cannot confirm restoration of disc structure or paraspinal tissue regeneration.

### Mechanisms underlying improvement in functional disability

Functional limitations are a core factor affecting the quality of life in DLDD patients. ODI evaluates patients' limitations in daily activities such as bending, walking, lifting, and prolonged sitting. This study showed that ODI scores at 4 weeks after treatment and at follow-up remained significantly lower in the observation group, indicating that PRP combination therapy not only alleviates pain but also promotes recovery of functional activities. This effect is not solely dependent on analgesia but is likely related to improved paraspinal muscle nutrition, enhanced soft tissue perfusion, and recovery of muscle endurance. Previous studies (26) have shown that bioactive factors in PRP can stimulate fibroblast and endothelial cell proliferation, enhance myofascial microcirculation, restore paraspinal muscle tone and elasticity, and improve trunk stability and movement coordination. Wongjarupong et al. (27) reported that a single PRP injection improved ODI mainly via pain reduction leading to indirect functional gains; in contrast, multiple injections combined with celecoxib in this study led to greater ODI improvement, further supporting that functional improvement may be related to combined anti-inflammatory and symptom-modulating effects. Kunal et al. (28) demonstrated that multiple PRP interventions improved dynamic musculoskeletal function within 12 weeks. These findings suggest the potential clinical relevance of PRP-based treatment, although the durability of functional improvement and its relationship with structural disc changes require further confirmation in studies with longer follow-up and imaging-based outcome assessment.

### Clinical application value and safety analysis

This study showed that the adverse reaction rate in the observation group was 14.29%, slightly higher than 8.93% in the control group, but the difference was not statistically significant. All reactions were transient local redness or soreness following injection, which resolved spontaneously within a short period, and no severe allergic reactions or infections were observed. Since PRP is derived from the patient's own blood and contains no exogenous components, its good biocompatibility results in low immunogenicity, making its safety profile prominent. This has also been confirmed in previous related studies (29, 30). Compared with the potential gastrointestinal discomfort, renal burden, or cardiovascular risks associated with long-term NSAID use, local PRP injection is a minimally invasive intervention that avoids systemic drug-related side effects. Additionally, PRP is simple to prepare, repeatable, and provides a potential adjunctive option for DLDD patients for whom conservative therapy has failed but surgery is not yet indicated. For pain and rehabilitation departments, the combined use of PRP and celecoxib may be useful for short-term symptom control and functional improvement. However, whether this strategy can delay disease progression or provide durable quality-of-life benefits requires confirmation in studies with longer follow-up.

### Study limitations and further prospects

Although this study provides preliminary clinical evidence for the combined use of autologous PRP injection and celecoxib in patients with DLDD, several limitations should be acknowledged. First, this was a retrospective single-center study with a relatively small sample size. Therefore, selection bias and residual confounding could not be completely avoided. In addition, treatment allocation was based on the therapy actually received in routine clinical practice rather than prospective randomization. Although the baseline characteristics were comparable between the two groups, unmeasured factors, including symptom severity, patient preference, physician decision-making, rehabilitation adherence, and previous conservative treatment history, may still have influenced treatment selection and clinical outcomes. Accordingly, the findings of this study should be interpreted as exploratory and should not be directly generalized to broader DLDD populations or to institutions with different PRP preparation, injection, and rehabilitation protocols.

Second, the follow-up duration was limited to 4 weeks after treatment. This short-term follow-up interval was mainly determined by the retrospective design and the availability of routinely documented clinical records. In our clinical practice, the first 4 weeks after completion of the initial treatment cycle represent the most consistently recorded period for evaluating early pain relief, functional recovery, inflammatory marker changes, and treatment-related adverse reactions. Therefore, the present study was designed to assess short-term clinical efficacy and early safety rather than long-term durability. A 4-week follow-up period is insufficient to determine whether PRP therapy provides sustained symptom control, reduces recurrence, delays disease progression, or promotes structural repair of the intervertebral disc. These long-term outcomes should be evaluated in future prospective studies with extended follow-up periods.

In addition, although the PRP preparation process in this study followed a consistent two-step centrifugation protocol, fixed injection volume, immediate use after preparation, and basic quality-control procedures, detailed PRP characterization was still limited. Because this was a retrospective study based on routine clinical records, post-preparation platelet concentration, leukocyte content, platelet activation status, and growth factor levels were not systematically quantified or consistently available for all patients. These factors may influence the biological activity of PRP and limit the reproducibility and comparability of the findings across studies.

Finally, this study mainly evaluated clinical symptoms, functional scores, inflammatory markers, and adverse reactions, but did not include MRI-based imaging outcomes, such as Pfirrmann grading or quantitative assessment of nucleus pulposus water content. Therefore, the present findings are insufficient to directly confirm structural regeneration of the intervertebral disc. Future multicenter, large-sample, prospective randomized controlled trials with longer follow-up periods are needed. These studies should incorporate standardized PRP preparation protocols, detailed PRP quality-control indicators, and imaging-based outcome measures to further verify the long-term efficacy, safety, and regenerative potential of PRP-based treatment for DLDD.

## Conclusion

The results of this study indicate that combining autologous PRP injection with oral celecoxib significantly enhances the overall efficacy in DLDD patients. Patients in the observation group showed superior outcomes in pain relief, lumbar function recovery, and improvement of daily functional activities compared with the control group receiving medication alone. Inflammatory markers such as TLR4, TNF-α, hs-CRP, and SDF-1 decreased significantly, suggesting that the observed short-term clinical and functional improvements may be partly associated with PRP-related modulation of inflammatory responses. The combined treatment had a low incidence of adverse reactions, mostly minor and transient, demonstrating high safety. In summary, autologous PRP injection as an adjunct to conservative DLDD therapy may provide short-term symptomatic relief, functional improvement, and favorable changes in inflammatory markers. However, because the follow-up duration was limited to 4 weeks, the present study cannot determine the long-term durability of PRP therapy or confirm structural regeneration of the intervertebral disc. Future multicenter, large-sample, long-term randomized controlled studies are warranted to further verify its sustained efficacy, safety, and standardized clinical application.

## Data Availability

The original contributions presented in the study are included in the article/supplementary material, further inquiries can be directed to the corresponding author.
